# Current Limitations and Perspectives in Single Port Surgery: Pros and Cons Laparo-Endoscopic Single-Site Surgery (LESS) for Renal Surgery

**DOI:** 10.1155/2010/759431

**Published:** 2010-02-14

**Authors:** Peter Weibl, Hans-Christoph Klingler, Tobias Klatte, Mesut Remzi

**Affiliations:** Department of Urology, Medical University of Vienna, AKH, Währinger Gürtel 18-20, 1090 Wien, Austria

## Abstract

Laparo-Endoscopic Single-Site surgery (LESS) for kidney diseases is quickly evolving and has a tendency to expand the urological armory of surgical techniques. However, we should not be overwhelmed by the surgical skills only and weight it against the basic clinical and oncological principles when compared to standard laparoscopy. The initial goal is to define the ideal candidates and ideal centers for LESS in the future. Modification of basic instruments in laparoscopy presumably cannot result in better functional and oncological outcomes, especially when the optimal working space is limited with the same arm movements. Single port surgery is considered minimally invasive laparoscopy; on the other hand, when using additional ports, it is no more single port, but hybrid traditional laparoscopy. Whether LESS is a superior or equally technique compared to traditional laparoscopy has to be proven by future prospective randomized trials.

## 1. Introduction

 Laparo-endoscopic single-site surgery (LESS) as a new alternative to conventional laparoscopy has gained popularity. Today laparoscopy has changed kidney surgery at all. Laparoscopic radical nephrectomy is gold standard when opting for radical nephrectomy in T1b-T2 renal cell cancer [EAU + AUA guidelines], but furthermore, laparoscopy is preferred for pyeloplasty and is comparable in nephron-sparing surgery for T1a renal tumors and in nephroureterectomy [1], some of them have also been described in the pediatric poplulation [2].

Various terms have been used for LESS up to date, but the final definition has been established in July 2008 by the Laparo-Endoscopic Single-Site Surgery Consortium for Assessment and Research (LESSCAR) as laparo-endoscopic single-site surgery (LESS) [3]. There are several important questions that should be answered until LESS will be equivalent with standard laparoscopy (SL). Is there any overall benefit for the patients in terms of risk of perioperative, postoperative morbidity, and oncological safety? 

Should we limit surgeons comfort and confidence? Which population of patients will actually benefit and what are the optimal indications? The aim of our minireview is to critically summarize pros and cons of LESS in renal surgery.

## 2. Potential Advantages and Disadvantages

 Although LESS is a rapidly evolving surgical minimally invasive technique, published reports are limited by numbers of patients and centers [1–9]. Meanwhile, it is very doubtful if LESS is going to further improve SL. Unproven potential advantages of single port surgery are only less scar, less discomfort, reduced postoperative pain, and thus less use of analgetic medication, followed with faster recovery and shorter hospital stay when compared to the traditional open and SL. LESS is feasible, with comparable perioperative and postoperative outcomes with limited follow up when compared to SL [1–11]. Beacause only the surgical technique itself has been modified, it is very uncertain that the oncological or clinical outcomes will be better than in SL. 

LESS creates a challenge for surgeons and increases their skills and ambidexterity. From our own initial experience, we think LESS is ideal for renal, adrenal cysts, cryoablation of small renal masses; however, we prefer the lower abdomen instead of the umbilicus for single port placement. Postoperative pain does not seem to be reduced compared to the SL surgical procedure (our unpublished data). We think that the overall benefit of LESS is lacking today. Even in high-volume laparoscopic centers like ours, the key issue will be right patient selection. 

The incision length varies usually from 1 to 6 cm [5, 6]. In SL for renal tumors, we use 2(1) 12 mm ports, 1(2) 5 mm port, and eventually another 5 or 12 mm port (overall length 34 mm). Of course an additional incision has to be made for organ extraction, but this is also true for LESS, unless natural orificions will be used or morcelleration like in the beginning of SL is used. The only difference is the range of few centimeters. Do we really have to measure the clinical equivalence of surgical procedure by cosmetics, or do we actually measure and compete ourselves as surgeons? The need is to critically evaluate this novel approach especially in patients with neoplasms. 

The maneuverability of instruments is more difficult in the single port platform, which might be overcome with the learning curve. Easier clashing of working instruments results in limited operating fields. Therefore, using an additional port is sometimes necessary [6]; others tend to insert percutaneously 3 mm small instruments [5], without adding an additional port and thus trying to fulfill the criteria of single port laparoscopy. Introduction of these advanced technologies and instruments (roticulating forces, flexible laparoscopic scissors, graspers) tends to overcome these limitations [3], which raises the question: is this modification of basic principle necessary?

Certainly those who will not perform many cases or at least on a regular basis, do not get better results.

## 3. Ideal Indications

 LESS is a challenging operation for an experienced laparoscopic surgeon [7]. It seems that in the future LESS will be equally efficocious and feasible to SL in high-volume centers. However, the main and probably the only advantage stays the single scar with potential increase in overall costs when compared to SL. 

Who will mainly benefit from LESS renal surgery: (1) patients who are most concerned of cosmesis, (2) nonextirpative surgeries such as renal, adrenal cyst marsupialization, pyeloplasty, renal tumor ablative techniques, or simple nephrectomy for small nonfunctioning kidney, (3) radical nephrectomy with morcelation where the lengthening of an incision is not necessary, which is on the other hand an oncological compromise and clearly will reduce postoperative oncological assessments. 

From our own experience, renal, adrenal cyst marsupialization and cryoblation of small renal mass were the ideal indications to start with comparable overall outcomes when compared to SL. Radical nephrectomy was feasible for an experienced laparoscopist equally in terms of perioperative and postoperative parameters as with SL. We have experienced two conversions due to adhesions in patients with previous abdominal surgery (unpublished data) to SL. Goel and Kaouk recommend cryoablation as an ideal procedure to start with single port surgery from their experience as well [12]. 

Patients with conventional contraindications to SL, previous ipsilateral renal surgery, or the presence of a solitary kidney should not be the candidates for LESS [8], at least initially or until the surgeon feels the same confidence as with SL. 

Partial nephrectomy remains to be very challenging even for laparoscopists in high-volume centers, with an experience over 950 SL partial nephrectomy cases. The major problem was the tissue retraction and therefore the ideal candidates would be nonobese, medium height with anterior exophytic lower pole tumor less than 4 cm with no previous abdominal surgery, with the possibility of extirpation without hilar clamping [7, 13]. 

In general SL has a higher ischemia time than open nephron-sparing surgery and thus has not reached the full competitive potential to open nephron-sparing surgery. That is why, LESS will certainly not reduce ischemia times, which is clearly a safety issue for the further kidney-function and the health of the patient.

Maybe LESS is a crossing bridge to the integration of LESS and robotics? What has been proved by Desai et al. in robot/assisted-LESS pyeloplasty, where other working instruments were inserted through separate fascial puncture, but through the umbilical incision [6]? It looks like a logical next step, because freedom of movement in robotic surgery eliminates basic limitations of this novel approach itself.

We do have to be critical to ourselves, because to date we can review a small volume of outcomes. As far as all these reports are initial, we should not expect the better outcomes, but comparable, what seems to be proven [1]. 

Last but not least, the overall rate of complications of laparoscopic procedures in urology is quite low (around 0.2%) [11]. Will be the “one scar LESS surgery” related to lower incidence of complications? Comparison of SL versus hand assisted laparoscopic renal surgery so far did not prove the fact that a smaller incision has a better outcome [9]. To date limited data on postoperative, port related morbidity, and cosmetics are still to be proven in comparative prospective trials. Surgeons are doctors at first and that is why novel techniques should not result in a race and competition in surgical minimalism.

## 4. Future Improvements of Less Technique

 Further technical improvements to minimize the invasiveness and upgrade LESS surgery are in progress. Magnetic anchoring and guidance system (MAGS) technology (by developing of magnetically controlled and anchored intracorporeal surgical instruments) seems to be a promising technique to facilitate and advance LESS surgery [14]. A generated magnetic field, as we can obtain in magnetic resonance imaging, is regarded as the least procedure that can be medically applied. One of the limitations of this procedure is that the extracorporeal electromagnetic control system is too large, that is why the size needs to be miniaturized. 

Introduction of da Vinci robotic platform in combination with single port surgery is encouraging and appears to overcome some limitations of single port laparoscopic surgery itself. It is beneficial especially during intracorporeal suturing by improving ergonomics. 

The second generation laparoscopic instruments and the upgraded generation of intuitive robotic systems are a must to achieve the potential goals of LESS technique. 

The smaller is the incision the greater need is for smaller instruments and robots. What does it mean for the future? Development of minirobots anchored intraabdominally through the specific platforms. We are already on the beginning of the minirobotic revolution and translation from mini invasive surgery to pure intracavitary surgery. The technical potential of “in vivo robots” has to be investigated, well defined, and established in the clinical field to eliminate the difficulties in LESS surgeries [15].

## 5. Conclusions

 LESS has a potential in reduction of postoperative pain and cosmetics, but should these benefits justify the use of single port surgery over traditional laparoscopy? One can presume that the modification of instruments of laparoscopic technique in general will not result in better clinical or even more in oncological outcomes. Certainly, we can not compete the fact that LESS is a challenging technique and increases the skills and ambidexterity of the surgeons. However, we should take LESS into account and weight against basic clinical and oncological principles. At the moment, the sufficient “yes” for LESS as a supreme technique over the traditional laparoscopy has to proven by future prospective randomized trials. We think that LESS will play a role, but a minor role in laparoscopic renal surgery.
